# (2*R*)-2-Benzene­sulfonamido-2-phenyl­ethanoic acid: a new monoclinic polymorph

**DOI:** 10.1107/S160053680903606X

**Published:** 2009-09-12

**Authors:** Islam Ullah Khan, Shahzad Sharif, Muhammad Nadeem Arshad, Ejaz, Muhammad Idrees

**Affiliations:** a Materials Chemistry Laboratory, Department of Chemistry, GC University, Lahore, Pakistan

## Abstract

In the title compound, C_14_H_13_NO_4_S, a sulfonamide derivative of phenyl glycine, the aromatic rings are inclined at a dihedral angle of 28.03 (12)°. In the crystal, O—H⋯O hydrogen bonds link the molecules into chains propagating in [100] and a weak C—H⋯O interaction cross-links the chains in the *c*-axis direction. In the previously published polymorph, the dihedral angle between the aromatic rings is 45.52 (18)° and the structure is stabilized by three different types of ring motif.

## Related literature

For related sulfonamide structures see: Arshad *et al.* (2008*a* ▶,*b* ▶, 2009 ▶). 
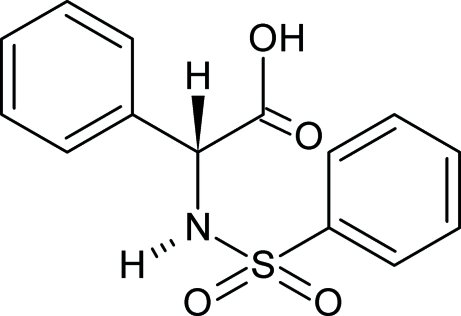


## Experimental

### 

#### Crystal data

C_14_H_13_NO_4_S
*M* *_r_* = 291.31
Monoclinic, 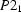

*a* = 8.2464 (8) Å
*b* = 5.3251 (4) Å
*c* = 15.3642 (15) Å
β = 100.384 (3)°
*V* = 663.64 (10) Å^3^
*Z* = 2
Mo *K*α radiation
μ = 0.26 mm^−1^
*T* = 296 K
0.34 × 0.19 × 0.11 mm

#### Data collection

Bruker Kappa APEXII CCD diffractometer
Absorption correction: multi-scan (*SADABS*; Bruker, 2007 ▶) *T*_min_ = 0.918, *T*_max_ = 0.972
7864 measured reflections
3174 independent reflections
2372 reflections with *I* > 2σ(*I*)
*R*_int_ = 0.034

#### Refinement

*R*[*F* ^2^ > 2σ(*F* ^2^)] = 0.042
*wR*(*F* ^2^) = 0.099
*S* = 1.01
3174 reflections
187 parameters
1 restraint
H atoms treated by a mixture of independent and constrained refinement
Δρ_max_ = 0.24 e Å^−3^
Δρ_min_ = −0.26 e Å^−3^
Absolute structure: Flack (1983), 1360 Friedel pairs
Flack parameter: −0.04 (9)

### 

Data collection: *APEX2* (Bruker, 2007 ▶ ▶); cell refinement: *SAINT* (Bruker, 2007 ▶ ▶); data reduction: *SAINT*; program(s) used to solve structure: *SHELXS97* (Sheldrick, 2008 ▶); program(s) used to refine structure: *SHELXL97* (Sheldrick, 2008 ▶); molecular graphics: *ORTEP-3 for Windows* (Farrugia, 1997 ▶) and *PLATON* (Spek, 2009 ▶); software used to prepare material for publication: *WinGX* (Farrugia, 1999 ▶) and *PLATON*.

## Supplementary Material

Crystal structure: contains datablocks I, global. DOI: 10.1107/S160053680903606X/hg2562sup1.cif

Structure factors: contains datablocks I. DOI: 10.1107/S160053680903606X/hg2562Isup2.hkl

Additional supplementary materials:  crystallographic information; 3D view; checkCIF report

## Figures and Tables

**Table 1:** Hydrogen-bond geometry (Å, °)

*D*—H⋯*A*	*D*—H	H⋯*A*	*D*⋯*A*	*D*—H⋯*A*
O4—H4*O*⋯O2^i^	0.83 (4)	1.98 (4)	2.727 (3)	150 (5)
C4—H4*A*⋯O3^ii^	0.93	2.56	3.317 (4)	139

Symmetry codes: (i) 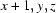; (ii) 
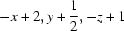
.

**Fig. 1.**
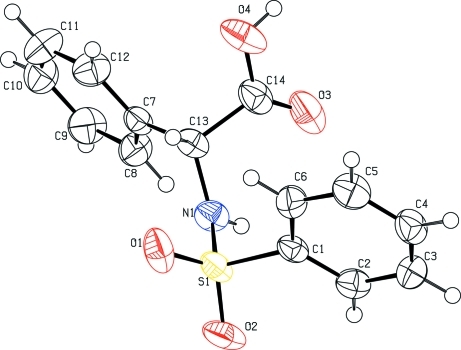
The labelled molecular structure diagram of the title compound with the 50% probability level of drawn thermal ellipsoids.

**Fig. 2.**
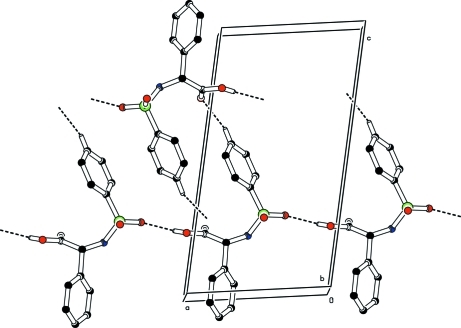
Unit cell packing diagram showing the intermolecular hydrogen bonding using dashed lines. The hydrogen atoms not involved in hydrogen bonding have been omitted.

## supplementary crystallographic information

### Comment

We have already reported the crystal structures of sulfonamides (Arshad *et al.*, 2008*a*, *b*), (Arshad *et al.*, 2009). In continuation of our studies in this area, we report here a new polymorph of our previously published sulfonamide (Arshad *et al.*, 2009a), derivative (II).

The title compound (I) crystallizes in the monoclinic space group P21. The molecule has a chiral center at C_13_ with a slightly distorted tetrahedral geometry. The dihedral angles between the two aromatic ring are 28.03 (12)° in (I) and 45.52 (18)° in (II). The crystal structure of I has no complex intermolecular interactions like II. There are only two types of hydrogen bonding interaction of O–H—O making a polymeric chain along the a-axes and C–H—O which linked these polymeric chain along c-axes (Fig. 2 and Table 1).

### Experimental

Phenyl glycine (2 g, 13.2 mmol) was dissolved in distilled water (15 ml) in a round bottom flask (50 ml). The pH of the solution was maintained at 8–9 using 1*M*, Na_2_CO_3_ solution. Benzene sulfonyl chloride (2.32 g, 13.2 mmol) was then suspended to the solution, and stirred at room temperature until all the suspension had been disappeared. On completion of the reaction the pH was adjusted 1–2, using 1 M HCl with stirring. The precipitate formed was filtered off, washed with distilled water, dried and recrystalized in methanol.

### Refinement

The H atoms for the C atoms were refined geometrically and treated as riding atoms: C—H = 0.93 for aromatic and C—H = 0.98 for the chiral carbon with *U*_iso_(H) = 1.2*U*_eq_. The N—H and O—H were refined in calculated positions and treated as riding atoms: O—H = 0.83 (4) Å, N—H = 0.82 (3) Å, with *U*_iso_(H) = 1.5*U*_eq_(parent O atom) and = 1.2*U*_eq_(parent N atom)

### Crystal data

**Table:**

C_14_H_13_NO_4_S	*F*(000) = 304
*M**_r_* = 291.31	*D*_x_ = 1.458 Mg m^−^^3^
Monoclinic, *P*2_1_	Mo *K*α radiation, λ = 0.71073 Å
Hall symbol: P 2yb	Cell parameters from 1972 reflections
*a* = 8.2464 (8) Å	θ = 2.5–23.4°
*b* = 5.3251 (4) Å	µ = 0.26 mm^−^^1^
*c* = 15.3642 (15) Å	*T* = 296 K
β = 100.384 (3)°	Needle, white
*V* = 663.64 (10) Å^3^	0.34 × 0.19 × 0.11 mm
*Z* = 2	

### Data collection

**Table:**

Bruker Kappa APEXII CCD diffractometer	3174 independent reflections
Radiation source: fine-focus sealed tube	2372 reflections with *I* > 2σ(*I*)
graphite	*R*_int_ = 0.034
φ and ω scans	θ_max_ = 28.3°, θ_min_ = 1.4°
Absorption correction: multi-scan (*SADABS*; Bruker, 2007)	*h* = −10→11
*T*_min_ = 0.918, *T*_max_ = 0.972	*k* = −7→6
7864 measured reflections	*l* = −20→20

### Refinement

**Table:**

Refinement on *F*^2^	Secondary atom site location: difference Fourier map
Least-squares matrix: full	Hydrogen site location: inferred from neighbouring sites
*R*[*F*^2^ > 2σ(*F*^2^)] = 0.042	H atoms treated by a mixture of independent and constrained refinement
*wR*(*F*^2^) = 0.099	*w* = 1/[σ^2^(*F*_o_^2^) + (0.0379*P*)^2^ + 0.1295*P*] where *P* = (*F*_o_^2^ + 2*F*_c_^2^)/3
*S* = 1.01	(Δ/σ)_max_ < 0.001
3174 reflections	Δρ_max_ = 0.24 e Å^−^^3^
187 parameters	Δρ_min_ = −0.26 e Å^−^^3^
1 restraint	Absolute structure: Flack (1983), 1360 Friedel pairs
Primary atom site location: structure-invariant direct methods	Flack parameter: −0.04 (9)

### Special details

**Table:**

Geometry. All e.s.d.'s (except the e.s.d. in the dihedral angle between two l.s. planes) are estimated using the full covariance matrix. The cell e.s.d.'s are taken into account individually in the estimation of e.s.d.'s in distances, angles and torsion angles; correlations between e.s.d.'s in cell parameters are only used when they are defined by crystal symmetry. An approximate (isotropic) treatment of cell e.s.d.'s is used for estimating e.s.d.'s involving l.s. planes.
Refinement. Refinement of *F*^2^ against ALL reflections. The weighted *R*-factor *wR* and goodness of fit *S* are based on *F*^2^, conventional *R*-factors *R* are based on *F*, with *F* set to zero for negative *F*^2^. The threshold expression of *F*^2^ > σ(*F*^2^) is used only for calculating *R*-factors(gt) *etc*. and is not relevant to the choice of reflections for refinement. *R*-factors based on *F*^2^ are statistically about twice as large as those based on *F*, and *R*- factors based on ALL data will be even larger.

### Fractional atomic coordinates and isotropic or equivalent isotropic displacement parameters (Å^2^)

**Table:**

	*x*	*y*	*z*	*U*_iso_*/*U*_eq_	
S1	0.53130 (7)	0.77328 (15)	0.29076 (4)	0.03965 (18)	
O1	0.5315 (3)	1.0355 (4)	0.27187 (13)	0.0569 (6)	
O2	0.3791 (2)	0.6402 (4)	0.28841 (13)	0.0560 (6)	
O3	0.9213 (3)	0.3901 (5)	0.25951 (16)	0.0728 (7)	
O4	1.0525 (2)	0.7469 (6)	0.23666 (16)	0.0700 (7)	
H4O	1.137 (5)	0.660 (9)	0.249 (3)	0.105*	
N1	0.6206 (3)	0.6352 (5)	0.21858 (15)	0.0396 (6)	
H1N	0.615 (4)	0.482 (6)	0.223 (2)	0.047*	
C1	0.6493 (3)	0.7278 (5)	0.39752 (16)	0.0334 (6)	
C2	0.6187 (3)	0.5204 (5)	0.44644 (19)	0.0411 (6)	
H2	0.5406	0.4020	0.4222	0.049*	
C3	0.7045 (3)	0.4914 (6)	0.53080 (19)	0.0448 (7)	
H3	0.6846	0.3519	0.5637	0.054*	
C4	0.8199 (3)	0.6657 (6)	0.56754 (19)	0.0454 (7)	
H4A	0.8754	0.6469	0.6255	0.054*	
C5	0.8524 (4)	0.8670 (6)	0.5181 (2)	0.0499 (8)	
H5	0.9327	0.9823	0.5422	0.060*	
C6	0.7673 (3)	0.9015 (6)	0.43262 (18)	0.0423 (6)	
H6	0.7893	1.0393	0.3995	0.051*	
C7	0.7556 (3)	0.7445 (5)	0.09274 (15)	0.0334 (5)	
C8	0.6714 (3)	0.5591 (6)	0.03958 (18)	0.0433 (7)	
H8	0.6165	0.4334	0.0647	0.052*	
C9	0.6688 (4)	0.5605 (6)	−0.0501 (2)	0.0518 (8)	
H9	0.6131	0.4341	−0.0852	0.062*	
C10	0.7472 (4)	0.7454 (7)	−0.08849 (19)	0.0540 (8)	
H10	0.7440	0.7458	−0.1493	0.065*	
C11	0.8301 (4)	0.9294 (7)	−0.0367 (2)	0.0601 (9)	
H11	0.8839	1.0552	−0.0624	0.072*	
C12	0.8347 (4)	0.9302 (6)	0.0532 (2)	0.0492 (7)	
H12	0.8915	1.0566	0.0877	0.059*	
C13	0.7667 (3)	0.7445 (6)	0.19245 (15)	0.0360 (6)	
H13	0.7771	0.9186	0.2133	0.043*	
C14	0.9209 (4)	0.6019 (6)	0.23465 (19)	0.0461 (7)	

### Atomic displacement parameters (Å^2^)

**Table:**

	*U*^11^	*U*^22^	*U*^33^	*U*^12^	*U*^13^	*U*^23^
S1	0.0295 (3)	0.0577 (4)	0.0306 (3)	0.0117 (3)	0.0025 (2)	−0.0032 (4)
O1	0.0695 (15)	0.0584 (14)	0.0409 (12)	0.0304 (11)	0.0050 (10)	0.0040 (10)
O2	0.0254 (9)	0.0997 (17)	0.0418 (12)	0.0027 (10)	0.0028 (8)	−0.0118 (11)
O3	0.0545 (14)	0.0755 (17)	0.0822 (18)	0.0204 (13)	−0.0038 (12)	0.0157 (14)
O4	0.0300 (10)	0.0916 (18)	0.0829 (17)	0.0032 (14)	−0.0042 (10)	−0.0082 (17)
N1	0.0373 (12)	0.0503 (13)	0.0324 (13)	0.0042 (11)	0.0096 (10)	−0.0076 (11)
C1	0.0263 (11)	0.0461 (17)	0.0274 (12)	0.0045 (12)	0.0038 (9)	−0.0056 (12)
C2	0.0339 (14)	0.0466 (16)	0.0425 (16)	−0.0035 (13)	0.0058 (12)	−0.0041 (13)
C3	0.0458 (16)	0.0509 (18)	0.0387 (16)	0.0017 (14)	0.0101 (13)	0.0087 (14)
C4	0.0434 (16)	0.0590 (19)	0.0317 (15)	0.0069 (14)	0.0013 (12)	−0.0001 (14)
C5	0.0445 (17)	0.0542 (18)	0.0465 (19)	−0.0095 (14)	−0.0036 (14)	−0.0098 (15)
C6	0.0412 (14)	0.0438 (14)	0.0405 (16)	−0.0008 (14)	0.0041 (12)	0.0043 (14)
C7	0.0277 (11)	0.0397 (15)	0.0324 (12)	0.0074 (12)	0.0041 (9)	−0.0005 (13)
C8	0.0457 (16)	0.0482 (16)	0.0344 (16)	−0.0072 (13)	0.0034 (13)	−0.0014 (13)
C9	0.0510 (18)	0.066 (2)	0.0370 (17)	−0.0071 (16)	0.0034 (14)	−0.0084 (16)
C10	0.0566 (16)	0.070 (2)	0.0360 (15)	0.0064 (19)	0.0107 (13)	0.0054 (17)
C11	0.064 (2)	0.066 (2)	0.056 (2)	−0.0083 (18)	0.0258 (17)	0.0109 (18)
C12	0.0486 (16)	0.0516 (18)	0.0479 (19)	−0.0066 (15)	0.0100 (14)	−0.0056 (15)
C13	0.0289 (11)	0.0439 (15)	0.0343 (13)	0.0034 (13)	0.0030 (9)	−0.0071 (13)
C14	0.0400 (16)	0.065 (2)	0.0310 (15)	0.0090 (15)	0.0002 (12)	−0.0056 (15)

### Geometric parameters (Å, °)

**Table:**

S1—O1	1.426 (2)	C5—C6	1.385 (4)
S1—O2	1.436 (2)	C5—H5	0.9300
S1—N1	1.615 (2)	C6—H6	0.9300
S1—C1	1.766 (2)	C7—C12	1.384 (4)
O3—C14	1.191 (4)	C7—C8	1.385 (4)
O4—C14	1.328 (4)	C7—C13	1.518 (3)
O4—H4O	0.83 (4)	C8—C9	1.375 (4)
N1—C13	1.458 (3)	C8—H8	0.9300
N1—H1N	0.82 (3)	C9—C10	1.368 (4)
C1—C6	1.380 (4)	C9—H9	0.9300
C1—C2	1.384 (4)	C10—C11	1.365 (5)
C2—C3	1.369 (4)	C10—H10	0.9300
C2—H2	0.9300	C11—C12	1.374 (4)
C3—C4	1.376 (4)	C11—H11	0.9300
C3—H3	0.9300	C12—H12	0.9300
C4—C5	1.368 (4)	C13—C14	1.522 (4)
C4—H4A	0.9300	C13—H13	0.9800
			
O1—S1—O2	120.73 (14)	C12—C7—C8	118.4 (3)
O1—S1—N1	106.78 (13)	C12—C7—C13	119.7 (2)
O2—S1—N1	105.24 (13)	C8—C7—C13	121.9 (2)
O1—S1—C1	107.61 (13)	C9—C8—C7	120.2 (3)
O2—S1—C1	106.75 (12)	C9—C8—H8	119.9
N1—S1—C1	109.41 (12)	C7—C8—H8	119.9
C14—O4—H4O	109 (3)	C10—C9—C8	120.8 (3)
C13—N1—S1	120.6 (2)	C10—C9—H9	119.6
C13—N1—H1N	119 (2)	C8—C9—H9	119.6
S1—N1—H1N	111 (2)	C11—C10—C9	119.4 (3)
C6—C1—C2	120.5 (2)	C11—C10—H10	120.3
C6—C1—S1	120.2 (2)	C9—C10—H10	120.3
C2—C1—S1	119.33 (19)	C10—C11—C12	120.5 (3)
C3—C2—C1	119.4 (3)	C10—C11—H11	119.7
C3—C2—H2	120.3	C12—C11—H11	119.7
C1—C2—H2	120.3	C11—C12—C7	120.6 (3)
C2—C3—C4	120.9 (3)	C11—C12—H12	119.7
C2—C3—H3	119.6	C7—C12—H12	119.7
C4—C3—H3	119.6	N1—C13—C7	112.0 (2)
C5—C4—C3	119.4 (3)	N1—C13—C14	110.6 (2)
C5—C4—H4A	120.3	C7—C13—C14	108.9 (2)
C3—C4—H4A	120.3	N1—C13—H13	108.4
C4—C5—C6	120.9 (3)	C7—C13—H13	108.4
C4—C5—H5	119.5	C14—C13—H13	108.4
C6—C5—H5	119.5	O3—C14—O4	126.0 (3)
C1—C6—C5	118.9 (3)	O3—C14—C13	124.3 (3)
C1—C6—H6	120.6	O4—C14—C13	109.7 (3)
C5—C6—H6	120.6		
			
O1—S1—N1—C13	40.7 (2)	C13—C7—C8—C9	−177.7 (3)
O2—S1—N1—C13	170.1 (2)	C7—C8—C9—C10	−0.8 (5)
C1—S1—N1—C13	−75.5 (2)	C8—C9—C10—C11	0.6 (5)
O1—S1—C1—C6	−23.6 (2)	C9—C10—C11—C12	−0.2 (5)
O2—S1—C1—C6	−154.5 (2)	C10—C11—C12—C7	0.1 (5)
N1—S1—C1—C6	92.1 (2)	C8—C7—C12—C11	−0.2 (4)
O1—S1—C1—C2	154.5 (2)	C13—C7—C12—C11	178.1 (3)
O2—S1—C1—C2	23.6 (2)	S1—N1—C13—C7	−133.6 (2)
N1—S1—C1—C2	−89.8 (2)	S1—N1—C13—C14	104.7 (2)
C6—C1—C2—C3	1.2 (4)	C12—C7—C13—N1	150.2 (2)
S1—C1—C2—C3	−176.9 (2)	C8—C7—C13—N1	−31.6 (4)
C1—C2—C3—C4	0.3 (4)	C12—C7—C13—C14	−87.1 (3)
C2—C3—C4—C5	−1.8 (4)	C8—C7—C13—C14	91.1 (3)
C3—C4—C5—C6	1.9 (5)	N1—C13—C14—O3	23.4 (4)
C2—C1—C6—C5	−1.1 (4)	C7—C13—C14—O3	−100.1 (3)
S1—C1—C6—C5	177.0 (2)	N1—C13—C14—O4	−158.5 (2)
C4—C5—C6—C1	−0.5 (4)	C7—C13—C14—O4	78.0 (3)
C12—C7—C8—C9	0.6 (4)		

### Hydrogen-bond geometry (Å, °)

**Table:**

*D*—H···*A*	*D*—H	H···*A*	*D*···*A*	*D*—H···*A*
O4—H4O···O2^i^	0.83 (4)	1.98 (4)	2.727 (3)	150 (5)
C4—H4A···O3^ii^	0.93	2.56	3.317 (4)	139

Symmetry codes: (i) *x*+1, *y*, *z*; (ii) −*x*+2, *y*+1/2, −*z*+1.
